# Interaction site prediction by structural similarity to neighboring clusters in protein-protein interaction networks

**DOI:** 10.1186/1471-2105-12-S1-S39

**Published:** 2011-02-15

**Authors:** Hiroyuki Monji, Satoshi Koizumi, Tomonobu Ozaki, Takenao Ohkawa

**Affiliations:** 1 Graduate School of System Informatics, Kobe University, Rokkodai, Nada, Kobe 657–8501, Japan; 2 Graduate School of Engineering, Kobe University, Rokkodai, Nada, Kobe 657–8501, Japan; 3 Cybermedia Center, Osaka University, Yamadaoka, Suita 565–0871, Japan

## Abstract

**Background:**

Recently, revealing the function of proteins with protein-protein interaction (PPI) networks is regarded as one of important issues in bioinformatics. With the development of experimental methods such as the yeast two-hybrid method, the data of protein interaction have been increasing extremely. Many databases dealing with these data comprehensively have been constructed and applied to analyzing PPI networks. However, few research on prediction interaction sites using both PPI networks and the 3D protein structures complementarily has explored.

**Results:**

We propose a method of predicting interaction sites in proteins with unknown function by using both of PPI networks and protein structures. For a protein with unknown function as a target, several clusters are extracted from the neighboring proteins based on their structural similarity. Then, interaction sites are predicted by extracting similar sites from the group of a protein cluster and the target protein. Moreover, the proposed method can improve the prediction accuracy by introducing repetitive prediction process.

**Conclusions:**

The proposed method has been applied to small scale dataset, then the effectiveness of the method has been confirmed. The challenge will now be to apply the method to large-scale datasets.

## Background

The functional analysis of proteins is an important issue for elucidating the mechanism of living bodies. Since most of the functions of proteins are largely-related to their 3D structures, research on estimating the function of protein by revealing the relation between the 3D structure and the function is one of the main stream of the structural bioinformatics.

Most of proteins express their functions by interacting with other proteins or ligands. In many cases, interaction occurs at local portion of a protein, which is called an *interaction site*. The structural and physical characteristics on the interaction site often determine the function of the protein, which means that clarifying the location of interaction site of the protein helps analyze the function of proteins.

Various methods for predicting interaction sites (or functionally significant sites) have been developed. Sacan et al. developed a tool for detecting family-specific local structural sites [1]. In their method, geometrically significant structural centers of the protein are detected, then features generated from the geometrical and biochemical environment around these centers are used to distinguish a family. Jones and Thornton proposed a method of predicting interaction sites by comparing the protein surface patches in terms of six properties [2]. In other approaches, interface residues in a protein are deduced by use of neural networks which have been trained with surface patches in protein structures and sequence profiles [3-6]. Support vector machines are also used in predicting interface residues [7-10]. Other methods involved in predicting interaction sites have been proposed [11].

Meanwhile, analyzing the function of proteins from the aspect of protein-protein interaction has gotten a lot of attention [12,13]. The development of experimental methods for observing interactions, such as the yeast two-hybrid, helps increase the data related to the protein-protein interaction, leading many databases [14-16], and is anticipated for understanding various biological phenomena [12,13,17]. Such data are mainly converted into the protein-protein interaction networks (PPI networks), which are often used as the protein function identification tools [18-21]. For example, six thousands of yeast genes library are used in creating protein-protein interaction map [18], several attempts analyzing over thousands kinds of protein-protein interactions have been addressed in full detail [19,20].

There is much research on identifying the function of proteins with PPI networks. Vazquez et al. have proposed the method predicting the function of protein nodes which are functionally unknown in PPI networks, and identifying the function of each node to optimize the function of whole nodes in the networks [12]. Also, they argue PPI networks are scalefree [22], which leads to many methods by probabilistic approach to complex networks. For example, Letovsky proposed a method for calculating probability of functional label given to nodes with propagation of the binomial model and Markov random field [13]. Deng et al. presented a protein function prediction method by assigning functions to all the unannotated proteins based on functions of the annotated proteins and the protein interaction network using Bayesian approaches [23].

In such PPI-based research, however, 3D protein structures are little considered. Since it is obvious that 3D protein structures make a strong contribution to the function of proteins, it must be significant to predict the interaction sites from the viewpoint of both the PPI networks and the 3D protein structures.

We propose a method of predicting interaction sites of a protein (target protein) whose structure has been solved but whose interaction site is unknown using the information of 3D structures and PPI networks. As it is known that the function of a protein is often similar to the function of neighboring proteins on the PPI network, interaction sites may be predicted by extracting pockets from the surface of the target protein whose shape and physical properties are similar to those of the neighboring proteins. However, the functions of all of the neighboring proteins are not always similar to the function of the target protein. Hence, the neighboring proteins are classified into several non-disjoint groups, each of which shares the common features based on structural similarity. The interaction sites are predicted by extracting common pockets that appear both in one of these groups and in the target protein. In addition, information of the neighboring proteins whose interaction sites have been specified by this method itself may be effectively utilized. That is, we assume that the predicted interaction site of the target protein is considered as a known interaction site, then the prediction process is repeated for other target proteins.

## Method

### Outline

**Figure 1:**
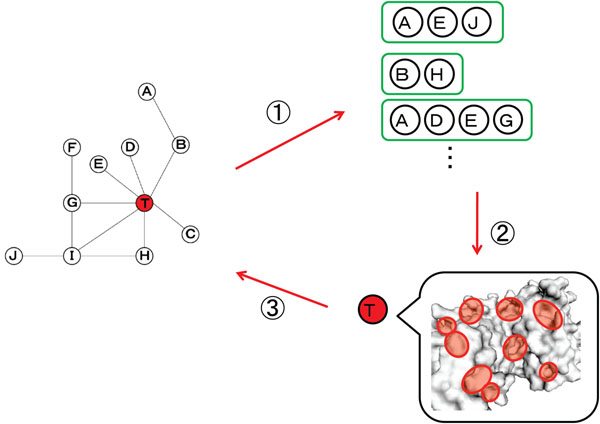
**Outline of the proposed method**. ‘*T*’ in red is a target protein and ‘*A*’ – ‘*J*’ indicate its neighboring proteins that are extracted from the PPI network, where the neighboring proteins are defined as proteins within a distance of two from the target protein in the PPI network. Interaction sites are predicted by extracting a pocket whose shape and physical properties are commonly observed among ‘*A*’ – ‘*J*’ and ‘*T*’.

Figure 1, in which ‘*T*’ in red is a target protein and ‘*A*’ – ‘*J*’ indicate its neighboring proteins that are extracted from the PPI network, shows the outline of prediction of interaction sites, where the neighboring proteins are defined as proteins within a distance of two from the target protein in the PPI network. Since the interaction site often forms a concave structure, instead of the whole of molecular surface of the protein, only pockets are treated as candidates of the interaction sites. In other words, interaction sites are predicted by extracting a pocket whose shape and physical properties are commonly observed among ‘*A*’ – ‘*J*’ and ‘*T*’. In practical cases, however, all of neighboring proteins ‘*A*’ – ‘*J*’ do not always have similar functions. For this reason, the groups, called neighboring protein clusters, in which a similar pocket is commonly observed, are extracted from ‘*A*’ – ‘*J*’. In our method, how to extract the cluster which shares discriminative pocket being similar in shape and physical properties is an important issue. If structurally similar groups are simply extracted from the neighboring proteins the cluster with similar structural features would be extracted, but the cluster which shares a “discriminative” pocket is not always obtained because the similarity of pockets which are observed in many proteins universally tend to be high. To cope with this problem, we introduce a restriction that each cluster must have at least one protein with known interaction sites. Next, the score is given for each pocket of the target protein which appears in all of extracted neighboring protein clusters commonly, and the top-ranked pockets are output as interaction sites.

Meanwhile, if the target protein ‘*T*’ has no neighboring protein with a known interaction site, it is impossible to construct any neighboring proteins clusters. To handle this difficulty, the prediction process is repeated by considering the predicted interaction sites as known interaction sites. In addition, repetition of the prediction process increases the neighboring proteins having the predicted (i.e. known) interaction sites, reorganization of the clusters using them will improve the prediction accuracy.

### Molecular surface data and pocket

In the proposed method, molecular surface data available from eF-site database [24] are used. A number of polygons represent the molecular surface, and every vertex composing polygons has the information of structure (location, maximum curvature, and minimum curvature), the property values (electrostatic potential and hydrophobicity), and the connection information of vertices. Interaction sites are widely known having concave structures on surface because of binding stability, specificity, and reaction promotion. Much research on searching and extracting pockets from the protein surface as candidates of interaction sites has been conducted [25,26].

In fact, the number of vertices of molecular surface of some proteins is over 20,000, so it is impractical idea to handle the whole molecular surface for comparing protein structures. Thus focusing on only pockets extracted from the molecular surface has advantages. In our method, the LIGSITE [27] algorithm is utilized to extract pockets. About 30 pockets are extracted for each protein.

### Representaion of pockets by histograms

It is known that proteins change their conformation in interacting, so comparing pockets by rigid superimposing of vertices which construct a pocket each other is inappropriate. So far, many methods for comparing surface patches have been proposed [28,29]. In order to compare molecular surfaces of the pockets from the viewpoint of mainly physical properties and roughly geometrical figures, we introduce a method of representing a molecular surface using histogram of structural and physical properties of the surface. Comparison of histogram is utilized in the area of such as image processing and it can compare pockets not definitely but roughly. As a pocket is constructed from vertex set of polygons, the pocket can be expressed with the four histograms, which are defined using three parameters, the range of rank *d*, the maximum value *max*, and the minimum value *min*, from four properties, namely, maximum curvature *κ_max_*, minimum curvature *κ_min_*, electrostatic potential *C*, and hydrophobicity *H*, of each vertex shown as follows. Values of the parameters *max*, *min*, and *d* are determined experimentally.

• Histogram of mean curvature:

*M* = (*κ_max_* + *κ_min_*)/2

*max* = 3.0, *min* = –3.0, *d* = 0.01

• Histogram of Gaussian curvature:

*G* = *κ_max_* · *κ_min_*

*max* = 3.0, *min* = –3.0, *d* = 0.01

• Histogram of electrostatic potential: *C*

*max* = 0.6, *min* = –0.6, *d* = 0.01

• Histogram of hydrophobicity: *H*

*max* = 5.0, *min* = –5.0, *d* = 0.1

### Similarity among pockets

A pocket is expressed using four histograms of structural and physical properties. We define similarity among pockets by comparing the four histograms.

Let *p*_1_,…,*p_N_* be *N* pockets and each pocket is expressed with the histogram of mean curvature *M_i_*(1 ≤ *i* ≤ *N*), the histogram of Gaussian curvature *G_i_*, the histogram of electrostatic potential *C_i_*, and the histogram of hydrophobicity *H_i_*. We simply define *S_pkt_*(*p*_1_, …, *p_N_*), the similarity among pockets *p*_1_,…,*p_N_*, by

*S_ptk_*(*p*_1_, …, *p_N_*) = *J*(*M*_1_,…,*M_N_*) × *J*(*G*_1_,…,*G_N_*) *× J*(*C*_1_,…,*C_N_*) *× J*(*H*_1_,…,*H_N_*) (1)

where *J*(*A*_1_,…,*A_N_*) represents the similarity among the histograms *A*_1_,…,*A_N_*, which is defined by(2)

where *a_i_k__*(1 ≤ *i* ≤ *N*) represents frequency of *k*-th rank of *i*-th histogram, and *n* represents the maximum value of the rank. Equation (2) is based on the idea of Jaccard coefficient to comparing histograms. That is to say, the similarity among pockets *S_pkt_* is defined as the product of the similarity of the four histograms expressing each pocket.

### Extraction of neighboring proteins cluster

In our method, we define a neighboring proteins cluster as a subset of proteins sharing the pockets that are similar in shape and physical properties and are specific to the cluster, which are extracted from the set of the neighboring proteins. We introduce the similarity measure that shows how similar the pockets on each protein in the subset are. If each protein in the subset has the similar interaction site, they are likely to share common pockets, then the similarity of the pockets in proteins in the subset must be high. Therefore, the pockets of each protein in the subset are exhaustively compared by using the similarity among pockets given by equation (1), then the highest similarity is put to be the subset similarity. However, there is a possibility that this highest similarity is actually due to the non-specific pockets which appear universally in the several proteins. To handle this matter, strong restriction is introduced, in which any subset must contain one or more proteins having a known interaction site. The following is an algorithm of extracting neighboring protein clusters.

1. Let P be a set of neighboring proteins, and S(⊆P) be a set of proteins in P whose interaction sites are known. *PS*(P, *n*) is a power set of P whose cardinality is *n*(1 <*n* ≤ *k*), and *ps* is an element of *PS*(P, *n*), namely *ps* ∈ *PS*(P, *n*). Enumerate all of *ps* satisfying the following constraint.

*ps* ∩ *S* ≠ *φ* (3)

2. Let *P*_*x*_1__, …, *P*_*x*_*n*__ be proteins in *ps*, namely *ps* = {*P*_*x*_1__,…,*P*_*x*_*n*__}. Calculate *S_set_*(*ps*), the similarity among {*P*_*x*_1__,…,*P*_*x*_*n*__} by the following definition

*S_set_*(*ps*) = *max*{*S_pkt_*(*p*^1^,…,*p^n^*)|*p^i^* ∈ *pkt*(*P_x_i__*), 1 ≤ *i* ≤ *n*} (4)

where *pkt*(*P_x_i__*) denotes a set of pockets in protein *P_x_i__*. *S_set_*(*ps*) means the similarity of the combination of the most similar pockets when exaustively comparing all the pockets of each protein.

3. Extract the elements ranked in the top *Z* of *PS*(P, *n*) as clusters *C* as follows.

*C* = {*x* ∈ *PS*(P, *n*)|*Rank*(*x*, *PS*(P, *n*)) <*Z*} (5)

where the function *Rank*(*x*, *PS*(P, *n*)) gives the ranking of *x* in *PS*(P, *n*) in terms of the similarity, which is formally defined as

*Rank*(*x*, *PS*(P, *n*)) *=* |{*y* ∈ *PS*(P, *n*)|*x* ≠ *y*, *S_set_*(*x*) <*S_set_*(*y*)}| (6)

**Figure 2:**
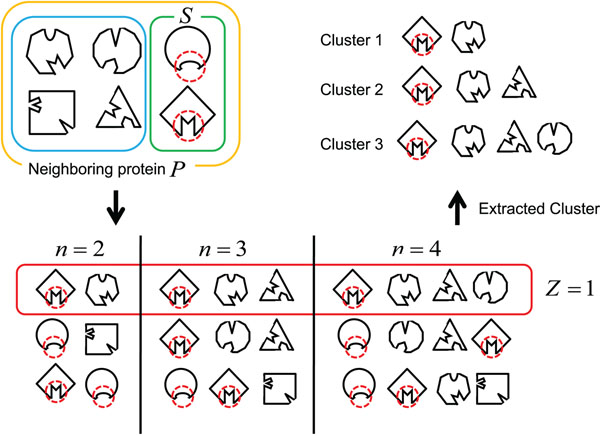
**Extraction of the neighboring proteins cluster**. A set of the neighboring proteins *P* is constructed from six proteins, and two of them are proteins having a known interaction site. The possible subsets of the neighboring proteins are enumerated, then they are ranked in accordance with the value of the similarity.

Figure 2 illustrates an example of extracting the neighboring protein clusters for *k* = 4 and *Z =* 1. In this example, a set of the neighboring proteins P is constructed from six proteins, and two of them are proteins having a known interaction site. *k* = 4 gives *ps*, the possible subset of the neighboring proteins set, whose size is 2, 3, or 4. First, *ps* which satisfies the constraint (3) is enumerated. Next, the enumerated *ps* is ranked in accordance with the value of the similarity. The constraint *Z =* 1 leads to extract *ps* with the highest similarity value as the clusters.

### Scoring of pockets

If a pocket in the target protein is similar to the pockets that appear commonly in the high ranked neighboring protein cluster, it may be a candidate of the interaction site. To evaluate each candidate, we introduce the voting-based scoring scheme. In this scheme, a set of pockets consisting of one pocket from the target protein and the similar pockets from the neighboring protein cluster is evaluated from the viewpoint of similarity, and the pocket (from the target) that wins the highest similarity value is voted. Formally, for a target protein *P_T_* and the proteins *P*_1_,…,*P_n_* in the neighboring protein cluster, the pockets to be voted are enumerated as follows.

*p* ∈ *pkt*(*P_T_*) s.t.

*S_pkt_*(*p*^1^,…,*p^n^*, *p*) = *S_set_*({*P*_1_,…,*P_n_*, *P_T_*}),

∀*i*, 1 ≤ *i* ≤ *n*, *p^i^* ∈ *pkt*(*P_i_*) (7)

### Complement and feedback by repetition

**Figure 3:**
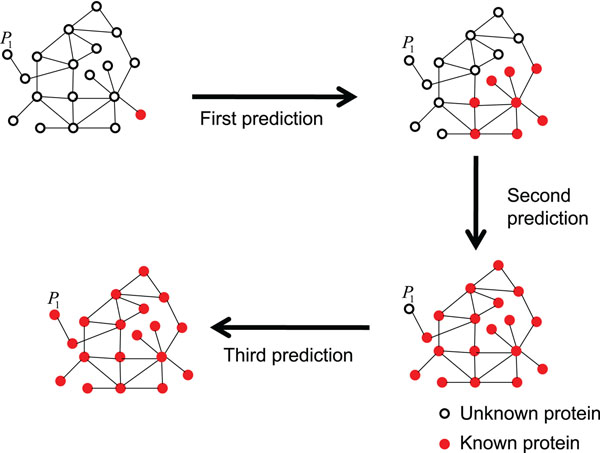
**Repetitive process of prediction.** There are few proteins that have known interaction sites in a single cycle prediction, but the repetitive prediction increases the proteins having known interaction site.

If the target protein has no neighboring proteins with the known interaction site, any cluster cannot be constructed because there is no *ps* satisfying the constraint (3). On the other hand, if the pockets of predicted protein are regarded as interaction sites we can get more proteins with known interaction sites on the PPI networks. That is, we can construct clusters using newly known (namely predicted) interaction sites, which enable repetitive prediction as shown in Figure 3. This repetitive process plays a complementary role for the protein which contains no interaction site in its neighboring proteins. Figure 3 shows that there are few proteins that have known interaction sites in a single cycle prediction, but the repetitive prediction increases the proteins having known interaction site.

Even if we deal in the target proteins whose neighboring proteins contain known interaction site, there is a possibility that the actual interaction site of the target protein may not be similar to any interaction sites of the neighboring proteins. The repetitive prediction plays a role of feedback for this problem. As the number of proteins whose neighboring proteins contain known interaction sites increases, the clusters of the neighboring proteins can be reconstructed. This feedback has the possibility to choose different pockets as interaction sites from the previous prediction process, and may get better result.

## Results and discussion

**Figure 4:**
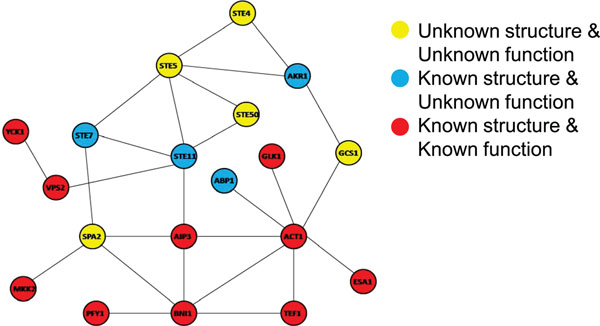
**Protein-protein interaction networks**. The red nodes are proteins having both structural and functional information, one of which is regarded as a prediction target by masking its functional information.

**Figure 5:**
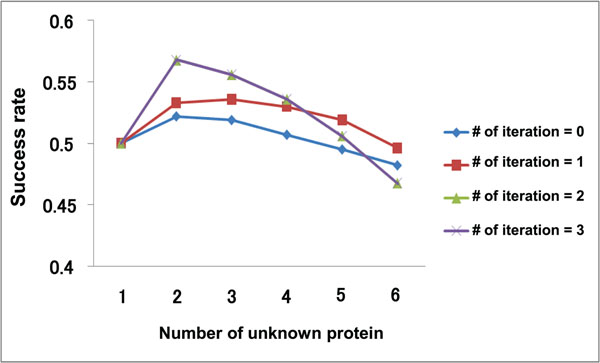
**The number of target proteins and the success rates(top)**. The result shows that the success rate in the repetitive predictions are usually higher than the case of predicting without repetition.

**Figure 6:**
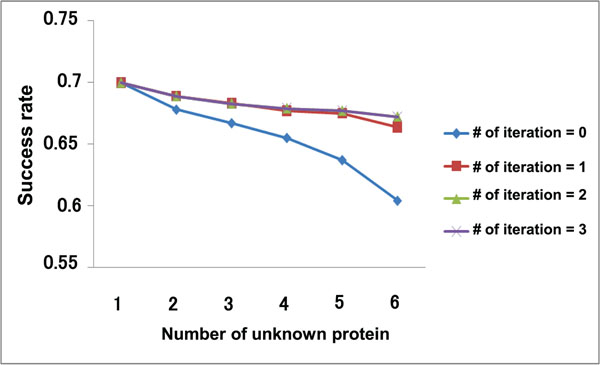
**The number of target proteins and the success rates(top five)**. The success rate gets better as the repetition count increases, and is converged at two times repetition regardless of the number of the target proteins.

To verify the effectiveness of the proposed method, we conducted experiments for prediction of interaction sites where we assumed that some of the known interaction sites are unknown. Figure 4 shows the PPI network used in these experiments. In Figure 4, *n* proteins are selected as target proteins, which mean that interaction sites are assumed to be unknown, from ten proteins whose interaction sites are actually known. Therefore, the interaction sites of *n* proteins are predicted at one cycle of the experiment. Furthermore, the *n* target proteins are combinatorially selected from the ten proteins (the number of combinations is _10_*C_n_*). We evaluate the results based on whether the pockets of the target proteins with the top-one score or the top-five score are true interaction sites. Figure 5 and 6 show the experimental results, in which the success rate in the case of top-one is about a half and in the case of top-five is about six or seven out of ten.

In addition, the result shows that the success rate in the repetitive predictions are usually higher than the case of predicting without repetition. This means the complement and the feedback by repetition work well. As for the effectiveness of the repetition process, Figure 6 shows the success rate gets better as the repetition count increases, and is converged at two times repetition regardless of the number of the target proteins. Figure 5 indicates that when the number of the target protein is large (i.e. *n* ≥ 5), the appropriate repetitive process contributes to rising the success rate, but repeating too much leads the decline of the success rate. It is considered that the first repetitive prediction improves the success rate by the effect of the complement. The feedback after the second repetitive prediction for a few of target proteins has a fine effect on success rate because of many neighboring proteins whose interaction sites have been known actually. In the case of a lot of target proteins are considered, however, the repetitive process may work worse because of the side effect of the feedback which are brought by the neighboring proteins having not actual but assumed interaction sites.

**Figure 7:**
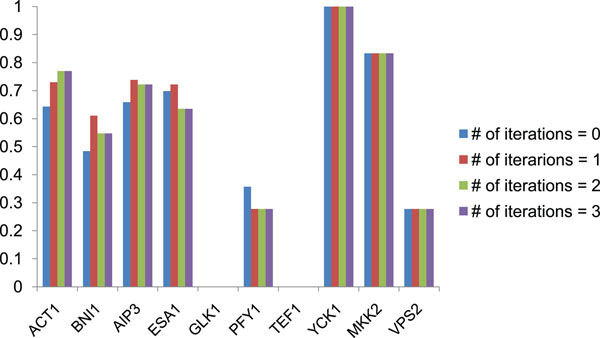
**Accuracy rates of predicting per proteins**. The success rate varies considerably depending on the protein. The success rate for proteins TEF1 and GLK1 is 0, so the interaction site cannot be specified at all.

**Figure 8:**
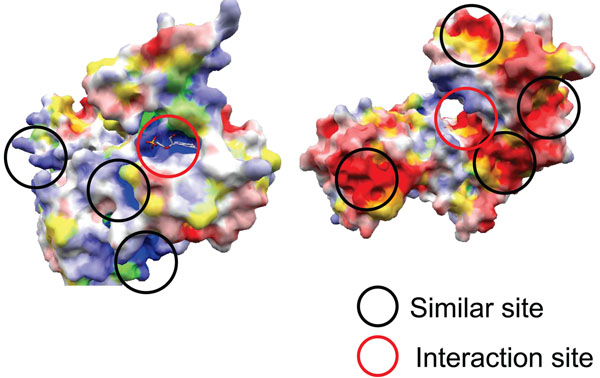
**Molecular surfaces of proteins (TEF1 and GLK1)**. The molecular surfaces of proteins TEF1 and GLK1 have a lot of local structures whose properties are similar to the true interaction sites, which might cause the prediction failure.

Figure 7 shows the success rates of prediction for each protein with *n* = 5 fixed. This figure means the success rate varies considerably depending on the protein. The success rate for proteins TEF1 and GLK1 is 0, so the interaction site cannot be specified at all. Figure 8 illustrates the molecular surfaces of TEF1 and GLK1. The molecular surfaces of these proteins have a lot of local structures whose properties are similar to the true interaction sites, which might cause the prediction failure. Our method assumes that the interaction sites have common structural features and share distinctive properties. This suggests that it is difficult to predict the interaction sites which have universal structures among proteins.

Finally we conducted comparative experiment, in which only structural information (i.e. without PPI networks) was utilized. In the basic situation, in which the number of target proteins is limited to one and no repetitive process is done, the success rate is 0.4, which is lower than the result by the proposed method (0.5). This result is under the limited situation only, but shows the effectiveness of the complementary use of the PPI network along with the protein structural information. We will perform comparative analysis for various experimental setting.

## Conclusions

We proposed a method of predicting interaction sites by comparing the pockets of proteins whose interaction sites are unknown to pockets of the neighboring proteins in the PPI networks. The challenge will be to apply this method to large-scale protein-protein interaction networks. In this paper, experimental results for the PPI networks consisting of only a few dozen of proteins have been presented. The nodes of actual PPI networks are, however, so massive that there is a need for experiments in larger networks. Now, therefore, it is desirable to apply the proposed method by dividing large-scale PPI networks into subgraphs comprising a few dozen of nodes. Currently, we put the strong restriction on our method, that is, we assume that the structural information of all prediction target proteins or neighboring proteins has been known. However, the method should be modified so that not only proteins with structural information but proteins having no structural information can be treated for practicality improvement. In addition, comparing accuracy of the proposed method to that of existing methods is a crucial remaining work in the near future.

## Competing interests

The authors declare that they have no competing interests.

## Authors' contributions

HM carried out the arrangement of the data set and the experimental results and drafted the manuscript. SK carried out the implementation of the algorithm and performed experiment. TO participated in the algorithm development and the design of the study. TO conceived of the study, and participated in its design and coordination and helped to draft the manuscript. All authors read and approved the final manuscript.
